# Inhibition of Nitric Oxide Synthase Prevents Muscarinic and Purinergic Functional Changes and Development of Cyclophosphamide-Induced Cystitis in the Rat

**DOI:** 10.1155/2014/359179

**Published:** 2014-06-01

**Authors:** Patrik Aronsson, Renata Vesela, Martin Johnsson, Yasin Tayem, Vladimir Wsol, Michael Winder, Gunnar Tobin

**Affiliations:** ^1^Department of Pharmacology, Institute of Neuroscience and Physiology, Sahlgrenska Academy, University of Gothenburg, P.O. Box 431, 40530 Gothenburg, Sweden; ^2^Department of Biochemical Sciences, Faculty of Pharmacy, Charles University, Heyrovskeho 1203, 50005 Hradec Kralove, Czech Republic; ^3^Department of Physiology and Pharmacology, Faculty of Medicine, Al-Quds University, P.O. Box 51000, Jerusalem, West Bank, Palestine

## Abstract

Nitric oxide (NO) has pivotal roles in cyclophosphamide- (CYP-) induced cystitis during which mucosal nitric oxide synthase (NOS) and muscarinic M5 receptor expressions are upregulated. In cystitis, urothelial muscarinic NO-linked effects hamper contractility. Therefore we wondered if a blockade of this axis also affects the induction of cystitis in the rat. Rats were pretreated with saline, the muscarinic receptor antagonist 4-DAMP (1 mg/kg ip), or the NOS inhibitor L-NAME (30 mg/kg ip) for five days. 60 h before the experiments the rats were treated with saline or CYP. Methacholine-, ATP-, and adenosine-evoked responses were smaller in preparations from CYP-treated rats than from saline-treated ones. Pretreatment with 4-DAMP did not change this relation, while pretreatment with L-NAME normalized the responses in the CYP-treated animals. The functional results were strengthened by the morphological observations; 4-DAMP pretreatment did not affect the parameters studied, namely, expression of muscarinic M5 receptors, P1A1 purinoceptors, mast cell distribution, or bladder wall enlargement. However, pretreatment with L-NAME attenuated the differences. Thus, the current study provides new insights into the complex mechanisms behind CYP-induced cystitis. The NO effects coupled to urothelial muscarinic receptors have a minor role in the development of cystitis. Inhibition of NOS may prevent the progression of cystitis.

## 1. Introduction

In rodents, as well as in humans, cyclophosphamide (CYP) treatment induces cystitis, which includes alterations both at functional and histological levels [1, 2]. Specifically, the urothelium/mucosa is affected, both regarding morphology and expression of receptors and signaling molecules. Functional changes occur via hampered efferent and afferent effects [3–6]. In conscious rats, this results in frequent micturitions of smaller volumes [7–9]. In the rat urinary bladder both acetylcholine and adenosine-5′-triphosphate (ATP) mediate the parasympathetic contractile response [10, 11]. In addition, the ATP metabolite adenosine evokes relaxations [12, 13]. The reduction of the parasympathetic contractile response in CYP-treated rats depends partly on an increased production of nitric oxide (NO) due to sensitization of urothelial muscarinic receptor stimulated NO effects [6, 14, 15]. The expression of nitric oxide synthase (NOS) in the mucosa has been reported to increase after CYP treatment [6, 16, 17]. CYP-induced morphological changes include bladder wall thickening [18], mast cell appearance in the smooth muscle [19], and upregulation of the expression of urothelial muscarinic M5 receptors [6, 20].

The CYP-induced cystitis in the rat is a commonly used disease model since it shares many features with the cystitis occurring in patients treated with CYP, but also with bladder pain syndrome/interstitial cystitis (BPS/IC) [21, 22]. BPS/IC is a noninfectious inflammatory condition with unclear etiology [23], including pelvic pain and voiding disturbances such as urinary urge and frequency. Although the pathophysiology of BPS/IC is largely unknown, it has been observed that the release of ATP and NO are associated with the condition [24, 25]. NO, in particular, is considered to have a pivotal role in this disease and mucosal NOS is upregulated in patients with BPS/IC corresponding to the findings in CYP-induced cystitis in the rat [6, 20]. Also, a pathognomonic mast cell infiltration into the detrusor muscle occurs [26–29]. Another factor suggested to be correlated to the degree of the disease is macrophage migration inhibitory factor (MIF) [30]. In CYP-induced cystitis in the rat increased levels of MIF occur in the urine and MIF can also be detected in all areas of the urothelium, not only in the basal part as is the case in healthy bladders [16].

We have recently demonstrated that pretreatment with the P1A1 antagonist DPCPX alleviate the symptoms to CYP-induced cystitis [31], and the aims of the current study were thus to examine if muscarinic receptors and NO are also involved in the inflammation in experimental CYP-induced cystitis, as well as to investigate if there exists a link between the two. Therefore, rats were pretreated with a NOS inhibitor or a muscarinic receptor antagonist before the induction of inflammation. Functional cholinergic and purinergic responses, tissue changes and the degree of mast cell infiltration, muscarinic M5 receptor, P1A1 purinoceptor, and MIF expressions were evaluated in saline and CYP-treated rats with particular care taken to the effects in the mucosa.

## 2. Materials and Methods

**Figure 1:**
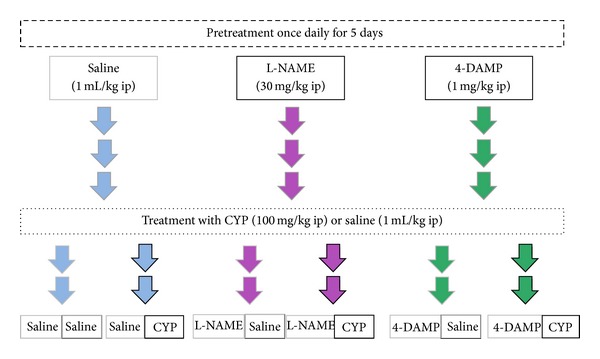
Study design. The rats were pretreated every morning for five days, starting at 120 hours before sacrifice. Sixty hours before the experiment, the rats received saline or CYP treatment and the rats were grouped into matched groups (saline-saline and saline-CYP, 4-DAMP-saline and 4-DAMP-CYP, L-NAME-saline and L-NAME-CYP; each group included 5-6 rats). Arrows indicate the five pretreatment events for each animal.

The ethics committee at the University of Gothenburg approved the study design, in which 38 male rats (300–400 g) of the Sprague-Dawley strain were used. For five days before sacrifice (starting at −120 h relative to sacrificing), the rats received daily intraperitoneal administrations of either saline, L-NAME (30 mg/kg), or 4-DAMP (1 mg/kg; Figure 1 shows a schematic layout of the study design). Cystitis was chemically induced (at −60 h relative to sacrificing) by a single intraperitoneal injection of CYP (100 mg/kg), while controls received the same volume of saline (9 mg/mL ip; 1 mL/kg). Both injections were conducted in the presence of the analgesic buprenorphine (10 *μ*g/kg ip). Sixty hours subsequent to the saline/CYP treatment (at the peak of inflammation [6]), the rats were anaesthetized with medetomidine (Domitor 0.3 mg/kg ip) then gassed and killed with an overdose of carbon dioxide. From the sacrificed rats, the urinary bladder was excised and thereafter divided into two identical parts; one half was used for functional examinations, while the other half was used for morphological and biochemical examinations.

### 2.1. In Vitro Functional Examinations

From the part of the bladder designated for functional examination, full thickness transverse strips were prepared (6 × 2 mm). The weights of the strips were 9.7 ± 0.3 (saline-saline; *n* = 10), 13.7 ± 0.5 (saline-CYP; *n* = 12), 14.0 ± 1.3 (4-DAMP-saline; *n* = 8), 19.0 ± 1.5 (4-DAMP-CYP; *P* < 0.001 versus saline-saline; *n* = 10), 12.5 ± 2.9 (L-NAME-saline; *n* = 8), and 16.2 ± 2.1 (L-NAME-CYP; *n* = 8) mg. The strips were excised from the detrusor proximal to the orifices of the two ureters in accordance to a standard procedure. Two strip preparations were taken from each bladder. The preparatory methods were generally as described previously for full thickness urinary bladder strip preparations with intact urothelium [12]. The detrusor strip was mounted between two steel rods of which one was fixed and the other adjustable and connected to an isometric force transducer (Linton). The strips were immersed in 25 mL organ baths containing Krebs solution of the following composition (mM): NaCl 118, KCl 4.6, CaCl_2_ 1.25, KH_2_PO_4_ 1.15, MgSO_4_ 1.15, NaHCO_3_ 25, and glucose 5.5, and which was gassed with 5% CO_2_ in O_2_ at 37°C. The preparations were repeatedly stretched in order to obtain a stable tension of about 5 mN after 45–60 min. The agonists utilized in the current studies, namely, methacholine (nonselective muscarinic agonist), ATP (nonselective P2 purinoceptor agonist), and adenosine (nonselective P1 purinoceptor agonist), were dissolved in distilled water and added to the organ baths at a volume of 125 *μ*L. The total amount of agonist did not exceed 3% of the organ bath volume. Data were recorded using a MP100WSW data acquisition system and Acquire software (Biopac). A high K^+^ solution (124 mM K^+^ obtained by exchanging Na^+^ for equimolar amounts of K^+^) was administered in the beginning of each experiment in order to assess the viability of each strip preparation. Strip preparations that did not respond to potassium were omitted (<15 mN). Before continuing with the experiment the organ baths were washed three times with normal Krebs solution and equilibrated for 10 min. Relaxations to adenosine were studied on urinary bladder strip preparations precontracted with a medium K^+^ solution (50 mM K^+^ obtained by exchanging Na^+^ for equimolar amounts of K^+^). The precontractile tensions were 18 ± 1 (saline-saline; *n* = 10), 16 ± 1 (saline-CYP; *n* = 12), 15 ± 1 (4-DAMP-saline; *n* = 8), 13 ± 1 (4-DAMP-CYP; *n* = 10; *P* < 0.05 to saline-saline and L-NAME-CYP), 16 ± 1 (L-NAME-saline; *n* = 8), and 18 ± 1 (L-NAME-CYP; *n* = 8) mN. The differences in precontractile values were not statistically significant within the groups analyzed in the Results section. All drug concentrations presented in the Results section are based on pilot experiments or on previous studies [32, 33].

### 2.2. Morphological Examinations

The bladder tissues not used in the functional examination were fixed in phosphate buffered paraformaldehyde (4%; pH 7.0) and then embedded in paraffin for further investigation by immunohistochemistry or staining with toluidine blue used as a mast cell marker.

### 2.3. Immunohistochemistry

Sections of the tissues were investigated by immunohistochemistry using subtype specific muscarinic antibodies for the M5 receptor, the P1A1 purinoceptor, and MIF. Four *μ*m sections were deparaffinized in xylene and rehydrated in ethanol. Any protein cross-linking that may have been induced by formalin was broken by incubation in citrate buffer (10 mM; pH 6.0), in a steamer at 95–100°C for 30 min. Further incubation in goat serum in PBS (5%, 1 h) was performed to block nonspecific background staining. Sections were subjected to primary polyclonal rabbit antibodies, diluted 1 : 100 in PBS containing 1% goat serum at 4°C overnight. The following day the samples were incubated with secondary antibody Alexa Fluor 488 goat anti-rabbit IgG (1 : 250 in PBS containing 1% goat serum) and rhodamine conjugated phalloidin (15 *μ*L/mL; dissolved in methanol 1 mg/1.5 mL). Finally, dehydration by ethanol was performed and the slices were mounted with Prolong gold antifade reagent with DAPI and were then viewed by microscopy (Eclipse 90i, Nikon, Tokyo, Japan). Photos were taken using camera DS-Fi1 (Nikon, Tokyo, Japan) and software NIS-Elements D 3.10 (Nikon, Tokyo, Japan). Negative controls were handled at the same occasion in the same fashion, but without addition of the primary antibody.

Although the antibodies are regarded by their manufacturers to be specific, cross-reactions with other peptides can never be ruled out and the resulting staining will hence be referred to as “P1A1-like,” “M5-like,” and “MIF-like.”

### 2.4. Toluidine Blue Staining

Sections of the paraffin embedded bladders (4 *μ*m) were deparaffinized by three 10 min intervals in 100% xylene and then rehydrated by serial incubations in 99.5% (2 × 5 min), 95% (5 min), 70% (5 min), and 50% (5 min) ethanol in water, followed by deionized water (10 min). Subsequently, they were stained in toluidine blue working solution (consisting of 20 mL of 1 g toluidine blue + 100 mL 70% ethanol; 180 mL of 1% sodium chloride in deionized water, pH = 2.3) for 2.5 min. Thereafter they were washed in deionized water for 4 × 30 sec and dehydrated through 10 dips in 95% ethanol, 10 dips in 99.5% ethanol, and 2 × 3 min in xylene.

### 2.5. Materials

The following substances were employed in the pretreatment of the rats and in the contraction experiments: acetyl-*β*-methylcholine chloride (methacholine), adenosine, adenosine-5′-triphosphate (ATP), 4-diphenylacetoxy-N-methylpiperidine methobromide (4-DAMP), N_*ω*_-nitro-L-arginine methyl ester HCl (L-NAME), and cyclophosphamide monohydrate (CYP). All these substances were purchased from Sigma-Aldrich, St Louis, MO, USA. Buprenorphine (Temgesic, Schering-Plough, Brussels, Belgium) and medetomidine (Domitor Vet., Orion Pharma, Espoo, Finland) were purchased from Apoteket Farmaci, Apoteket AB, Stockholm, Sweden.

The following substances were used for the immunohistochemistry: Alexa Fluor 488 goat anti-rabbit IgG (purchased from Molecular Probes, Eugene, Oregon, US), rabbit polyclonal muscarinic M5 receptor antibody (Research and Diagnostic Antibodies, Berkley, US), ABC Staining system sc-2018 for use with rabbit primary antibody (Santa Cruz Biotechnology, USA), prolong gold antifade reagent with DAPI and PBS tablets (Invitrogen, Burlington, Canada), xylene (Histolab, Gothenburg, Sweden), and ethanol (Kemetyl, Stockholm, Sweden). Rabbit polyclonal P1A1 purinoceptor antibody, goat serum, rhodamine conjugated phalloidin, rabbit MIF antibody (Sigma-Aldrich, St Louis, USA), citrate buffer (citric acid and sodium citrate tribasic dehydrate), and methanol were all purchased from Sigma-Aldrich, St. Louis, MO, USA.

The following substances were used in the mast cell staining: hydrochloric acid, sodium chloride and toluidine blue O (Sigma-Aldrich, St Louis, USA), ethanol 99.5% and 95% (Kemetyl, Haninge, Sweden), pertex and xylene (Histolab Products AB, Gothenburg, Sweden), and phosphate buffered paraformaldehyde 4% (Apoteket Produktion & Laboratorier AB, Gothenburg, Sweden).

### 2.6. Calculations and Statistics

Statistical significance was determined by Student's* t*-test for unpaired data. When multiple comparisons with the same variable were made, statistical significance was determined by two-way analysis of variance or one-way analysis of variance (ANOVA) followed by the Bonferroni correction. Pharmacodynamic modeling was performed by applying built-in models (log (agonist) versus response (variable slope), biphasic or bell-shaped equations) for nonlinear and linear regression (GraphPad Software Inc., San Diego, USA). In the model fitting procedure, comparisons were based on visual inspection of observed versus predicted plots, value of the objective function, assessment of parameter correlation precision, and the Akaike information criterion. The measurements of bladder wall thicknesses were performed at four randomly chosen places at each specimen, and the average value was employed in the analyses. In the assessment of mast cell occurrence, all mast cells occurring in the whole half bladder segment (the half separated for morphological examination) were counted except for those in the serosa. *P* values of 0.05 or less were regarded as statistically significant. Data are presented as mean ± SEM. Graphs were generated and parameters computed using the GraphPad Prism program (GraphPad Software Inc., San Diego, USA).

## 3. Results

### 3.1. In Vitro Contractile Responses

**Figure 2:**
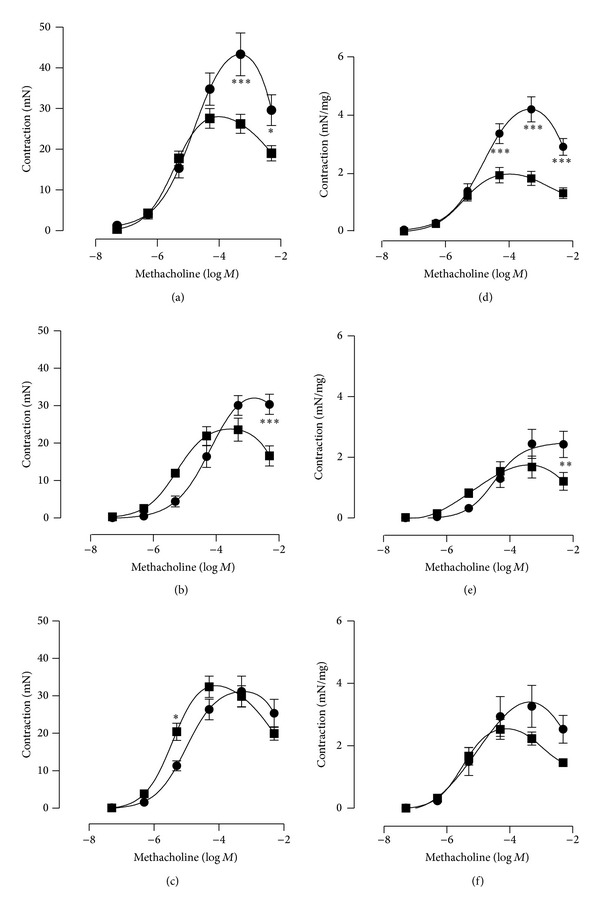
Mean contractions to methacholine of isolated urinary bladder strip preparations from control (saline-treated; ●) rats and rats with CYP-induced cystitis (■), pretreated with saline ((a), (d); *n* = 10 and 12), 4-DAMP ((b), (e); *n* = 8 and 10) or L-NAME ((c), (f); *n* = 8 in each group). The left column ((a)–(c)) expresses data as absolute values (mN) and the right column ((d)–(f)) as mN/mg. The vertical bars represent the SEM. ^∗^ denotes *P* < 0.05, ^∗∗^ *P* < 0.01, and ^∗∗∗^ *P* < 0.001.

**Figure 3:**
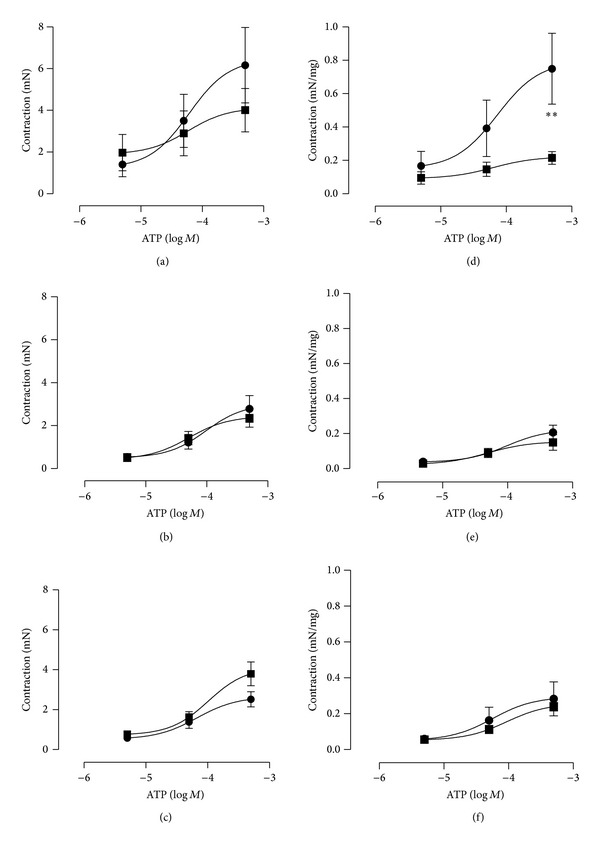
Mean contractions to ATP of isolated urinary bladder strip preparations from control (saline-treated; ●) rats and rats with CYP-induced cystitis (■), pretreated with saline ((a), (d); *n* = 10 and 12), 4-DAMP ((b), (e); *n* = 8 and 10) or L-NAME ((c), (f); *n* = 8 in each group). The left column ((a)–(c)) expresses data as absolute values (mN) and the right column ((d)–(f)) as mN/mg. The vertical bars represent the SEM. ^∗∗^ denotes *P* < 0.01.

**Figure 4:**
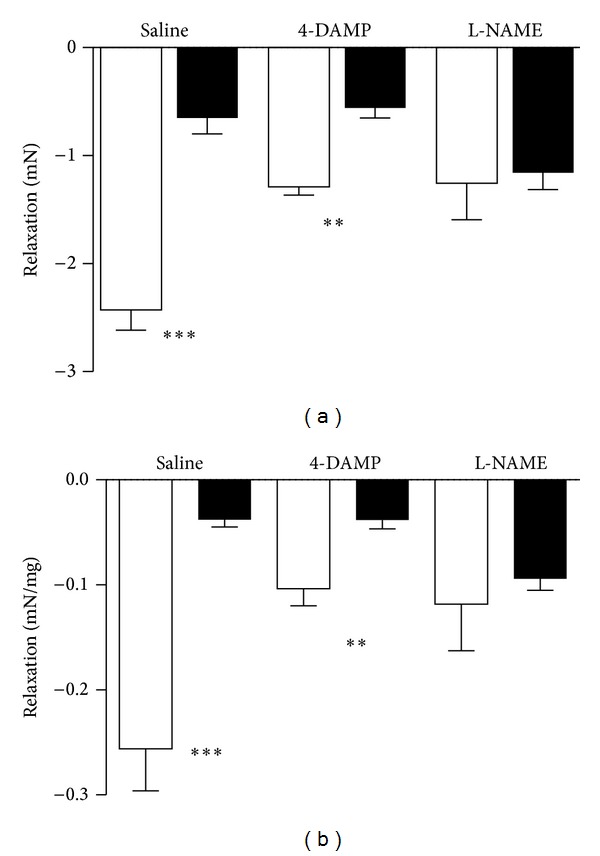
Mean relaxations to adenosine (5 × 10^−5^ M) of precontracted (K^+^; 50 mM) isolated urinary bladder strip preparations from control (saline-treated; white) rats and rats with CYP-induced cystitis (black), pretreated with saline (*n* = 10 and 12), 4-DAMP (*n* = 8 and 10), or L-NAME (*n* = 8 in each group). (a) expresses data as absolute values (mN) and (b) as mN/mg. The vertical bars represent the SEM. ^∗∗^ denotes *P* < 0.01 and ^∗∗∗^ *P* < 0.001.

Methacholine (5 × 10^−8^–5 × 10^−3^ M) evoked concentration-dependent contractions in saline- (normal and controls) and CYP-treated rats (Figure 2(a)). In the rats, which had only received saline as pretreatment (in contrast to L-NAME or 4-DAMP pretreatments), CYP-treatment (60 h before experiment) reduced the contractions. In saline-treated rats, the maximum methacholine response was 43 ± 8 mN (4.2 ± 0.4 mN/mg strip weight; 5 × 10^−4^ M; *n* = 10), whereas the maximum response was 26 ± 3 mN in CYP-treated rats (1.8 ± 0.2 mN/mg strip weight; 5 × 10^−5^ M; *P* < 0.001; *n* = 12; Figure 2(d)). The pEC50 values were 4.80 and 5.43 in saline pretreated and saline-treated rats and saline pretreated and CYP-treated rats, respectively (Table 1). The corresponding responses to methacholine at 5 × 10^−6^ M (close to EC_50_) were 15 ± 2 and 18 ± 2 mN, in saline and CYP-treated animals, respectively. Administration of Krebs solution with a high concentration of potassium (124 mM) tended to evoke larger contractions in saline-treated (36 ± 5 mN; *n* = 10) than in CYP-treated rats (30 ± 4 mN; n.s.; both pretreated with saline; *n* = 12). Likewise, the ATP-evoked (5 × 10^−6^–5 × 10^−4^ M) contractions seemed to be larger in the saline-treated bladders than in the CYP-treated (6 ± 2 (*n* = 10) versus 4 ± 1 mN (*n* = 12); Figure 3(a)), but no statistical significance was attained. When expressed as contraction per mg of tissue, the response was significantly larger in the saline-treated than in the CYP-treated group at 5 × 10^−4^ M ATP (0.75 ± 0.21 and 0.22 ± 0.04 nN/mg, respectively; *P* < 0.01, Figure 3(d)). The adenosine-evoked (5 × 10^−5^ M) relaxations to precontracted (K^+^; 50 mM) strip preparations were significantly larger in the saline-treated bladders than in the CYP-treated ones (−2.4 ± 0.1 (*n* = 10) versus −0.7 ± 0.1 (*n* = 12) mN; −0.26 ± 0.04 versus −0.04 ± 0.01 mN/mg; *P* < 0.001; Figure 4).

Pretreatment with the muscarinic receptor antagonist 4-DAMP (1 mg/kg ip) for five days (saline/CYP 60 h before experiment) did not change the relation between the maximum responses in the two groups of rats (maximum response in saline- and CYP-treated: 32 ± 2 (*n* = 8) and 24 ± 3 (*n* = 10) mN, respectively; *P* < 0.05; Figure 2(b)). At methacholine concentrations less than 10^−4^ M, the CYP-treated preparations seemed to be somewhat more sensitive than the saline-treated. However, no statistical significance was attained. The responses to 5 × 10^−6^ M of methacholine were 4 ± 2 and 12 ± 1 mN, respectively. Also the contractile responses to ATP showed great resemblance after the 4-DAMP pretreatment (2.8 ± 0.6 (*n* = 8) versus 2.4 ± 0.4 (*n* = 10) mN; n.s.; Figure 3(b)), while the adenosine relaxations were still different (−1.3 ± 0.1 (*n* = 8) versus −0.5 ± 0.1 (*n* = 10) mN; *P* < 0.01; Figure 4). However, pretreatment with the NOS inhibitor L-NAME (30 mg/kg ip) normalized the cholinergic contractile response in the CYP-treated animals (Figure 2(c)). Almost identical contractile responses to methacholine appeared in the two groups (maximum response in saline- and CYP-treated: 31 ± 4 and 34 ± 2 mN, respectively; n.s.; *n* = 8 in each group). At 5 × 10^−6^ M, the response to methacholine was even significantly larger in the CYP-treated rats than in control (20 ± 2 and 11 ± 1 mN, respectively; *P* < 0.05). Also, the Hill coefficients were close to 1 in both groups (Table 1). No significant differences occurred regarding the ATP contractions after the L-NAME pretreatments (2.5 ± 0.4 versus 3.8 ± 0.6 mN; n.s.; *n* = 8 in each group; Figure 3(c)). The adenosine-evoked relaxations were almost identical in the saline- and CYP-treated bladders after L-NAME pretreatment (−1.3 ± 0.3 versus −1.2 ± 0.2 mN; n.s.; *n* = 8; Figure 4).

The responses to potassium (124 mM) were still smaller, if anything, in the 4-DAMP pretreated CYP-treated rats than in the saline-treated (29 ± 2 (*n* = 8) versus 23 ± 4 (*n* = 10) mN; n.s.). In the L-NAME pretreated rats, the response in the saline-treated group was 25 ± 3 mN and in the CYP-treated group 26 ± 2 mN (*n* = 8 in each group; n.s.). The comparison of the overall maximum contractile responses to methacholine showed no significant differences within neither the saline-treated groups (43 ± 8 (*n* = 10), 32 ± 2 (*n* = 8) and 31 ± 4 (*n* = 8) mN; saline, 4-DAMP and L-NAME pre-treatment, resp.) nor the CYP-treated groups (26 ± 3 (*n* = 12), 24 ± 3 (*n* = 10) and 34 ± 2 (*n* = 8) mN, saline, 4-DAMP and L-NAME pretreatment, resp.), although the methacholine responses tended to be smaller after 4-DAMP and L-NAME pretreatments in saline-treated rats, and to be larger in the L-NAME pretreated group in CYP-treated rats. Neither did the overall maximum contractile responses to ATP differ significantly in the saline-treated group (5.5 ± 1.8 (*n* = 10), 2.5 ± 0.6 (*n* = 8) and 2.2 ± 0.5 (*n* = 8) mN; saline, 4-DAMP and L-NAME pre-treatment, resp.) nor did they differ in the CYP-treated (4.0 ± 1.0 (*n* = 12), 2.4 ± 0.6 (*n* = 10) and 3.8 ± 0.6 (*n* = 8) mN; saline, 4-DAMP and L-NAME pre-treatment, resp.) groups. However, the adenosine relaxations were significantly larger in the saline-treated and saline pretreated group than in the 4-DAMP and L-NAME pretreated groups (*P* < 0.01 (4-DAMP) and *P* < 0.001 (L-NAME)). The corresponding responses in the CYP-pretreated groups were not significantly different.

### 3.2. Morphological Examinations

**Figure 5:**
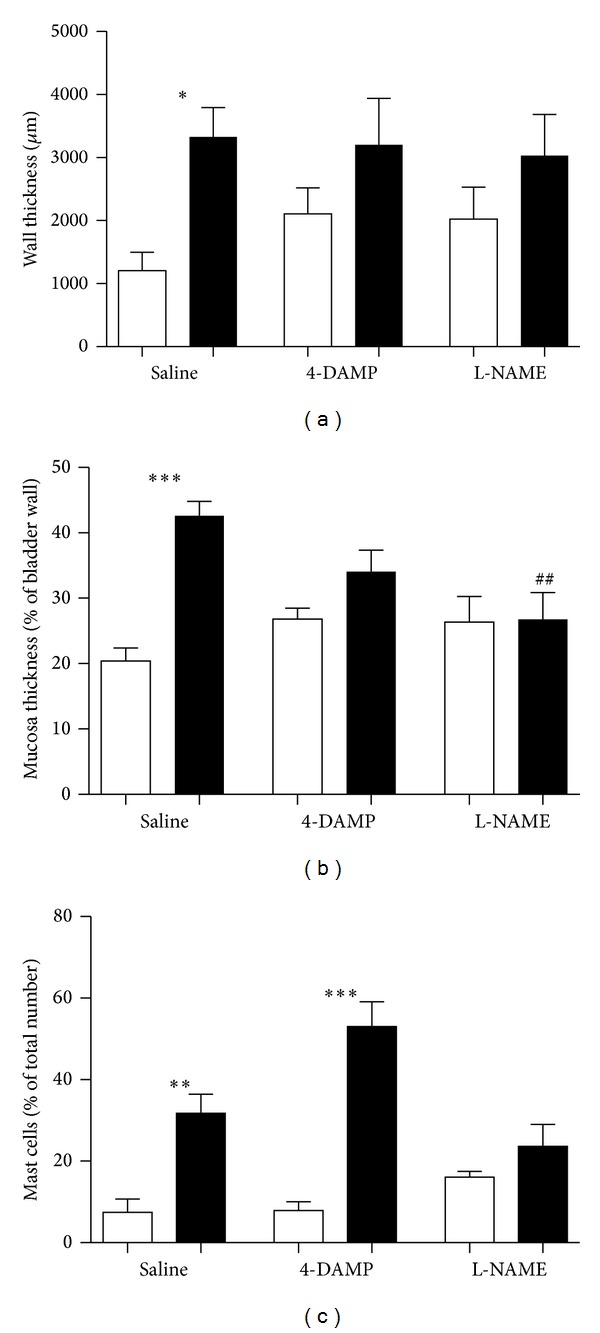
Bladder wall thickness (a), mucosa thickness (b), and ratio of mast cell occurrence in the detrusor (c), expressed as % of total number of mast cells (*n* = 3 in all groups) in saline-treated (white) and CYP-treated (black) rats. The vertical bars represent the SEM. ^∗^ denotes *P* < 0.05, ^∗∗^ *P* < 0.01 and ^∗∗∗^ *P* < 0.001 (comparisons between saline- and CYP-treated groups). ## denotes *P* < 0.01 (comparison with saline pretreated and CYP-treated group).

**Figure 6:**
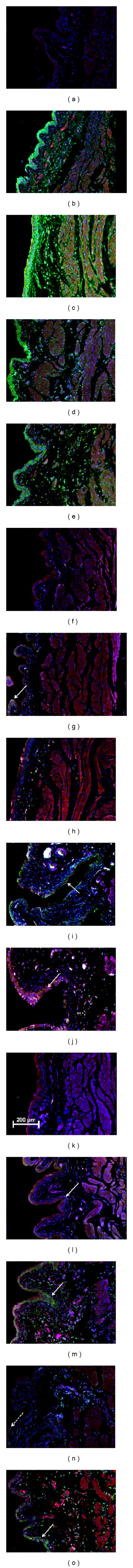
Representative images of immunohistochemical muscarinic M5 receptor-like (upper row; (a), (b), (c), (d), and (e)), P1A1 purinoceptor-like (middle row; (f), (g), (h), (i), and (j); solid arrows indicate staining) and MIF-like (lower row; (k), (l), (m), (n), and (o); solid arrows indicate staining, dashed arrow indicates possibly occurring staining) staining. Left columns show saline pretreated and saline treated (control), negative ((a), (f), (k)) and positive staining ((b), (g), and (l)). The middle column ((c), (h), and (m)) shows saline pretreated and CYP treated and the second column from the right ((d), (i), and (n)) shows L-NAME pretreated and saline treated bladders. The right column ((e), (j), and (o)) shows staining from L-NAME pretreated and CYP treated bladders. The horizontal bar represents 200 *μ*m for all images. All sections are counterstained with DAPI (blue) and phalloidin (red). Green color represents positive staining.

The inflamed bladder specimens showed a dramatic thickening of the bladder wall in comparison to the normal, saline-treated specimens. In urinary bladders from saline pretreated and saline-treated rats, the total wall thickness was 1.2 ± 0.3 mm, while in the corresponding CYP-treated group the wall thickness was 3.3 ± 0.5 mm (*P* < 0.05; *n* = 5; Figure 5(a)). Particularly, the urothelium/mucosa was enlarged, which is reflected by the mucosa versus total wall thickness ratio demonstrated in Figure 5(b) (20.4 ± 2.0 versus 42.6 ± 2.2% in saline-treated and CYP-treated groups, respectively; *P* < 0.001; *n* = 5). Also, the urothelium was less folded in the bladders from CYP-treated rats (Figure 6). In the muscularis layer, more stromal structures appeared and the muscle seemed less well organized. Both 4-DAMP and L-NAME possibly induced a slight bladder thickening in the saline-treated animals, however, statistical significance was not attained. No effects by the 4-DAMP and L-NAME pretreatments were observed on the total bladder wall thickness in the CYP-treated animals. However, the L-NAME pretreatment caused the CYP-treatment to induce less thickening of the mucosa (42.6 ± 2.2 versus 26.8 ± 4.1%; *P* < 0.01; *n* = 5; Figure 5(b)). Also the 4-DAMP pretreatment may possibly had prevented some of the urothelial/mucosal enlargement (34.1 ± 3.3%; n.s.; *n* = 5).

### 3.3. Mast Cell Infiltration

No differences in the total number of mast cells in the half bladder specimen (the assessment included the mucosa and the muscularis layers and not the serosa/adventitia) could be detected between the groups, but the variations between the individual rats were large (17–71 mast cells). However, the mast cells were differently distributed in the bladders of saline- and CYP-treated rats. In the saline-treated rats, only few mast cells occurred in the muscularis layer, which was reflected by the ratio of the number of mast cells in the muscularis expressed as percentage of the total number of mast cells in the bladder half. In the CYP-treated rats, there was a 4.5-fold increase of the ratio in comparison to saline-treated rats (32 ± 5 versus 7 ± 3%, *P* < 0.01; Table 2, Figure 5(c)). In the 4-DAMP pretreated animals, the ratio was even higher in the CYP-treated rats (6.5-fold; *P* < 0.001). However, the L-NAME pretreatment eliminated the difference in mast cell occurrence between saline- and CYP-treated rats.

### 3.4. Immunohistochemistry

In rats that had received saline pretreatment, the mucosal muscarinic M5 receptor-like staining was obvious in CYP-treated rats (Table 2, Figure 6). The specimens of saline pretreated and saline-treated rats showed little staining for muscarinic M5 receptor-like reactivity. Pretreatment with 4-DAMP did not affect the expression, neither in the normal nor in the cystitis group. However, pretreatment with L-NAME eliminated the difference in mucosal muscarinic M5 receptor-like expression between the saline- and CYP-treated groups. P1A1 purinoceptor-like expression showed little variance between saline-treated and CYP-treated bladders but tended to be less in the CYP-treated if anything. While 4-DAMP pretreatment had no effect on the expression, the L-NAME pretreatment may possibly have prevented some part of the CYP-induced P1A1 purinoceptor-like expression decrease. MIF-like staining occurred in both CYP-treated and saline-treated rats, which also showed a substantial variation within each groups. The staining seemed to occur more superficially in the urothelium in the tissue from CYP-treated rats. Neither 4-DAMP nor L-NAME pretreatment seemed to affect the MIF-like expression.

## 4. Discussion

Noxious stimuli to a tissue induce a battery of effects in which mediation of a number of different signaling molecules participates. In CYP-induced cystitis, urothelial NO and ATP play pivotal roles [24, 34, 35]. While the focus for the former has been in the aspect of inflammation progression, the latter has been discussed in relation to sensory mechanism. Consequently, it may be of clinical relevance to determine if inhibition of NOS generally impedes the induction of cystitis or just affects some parts of the inflammation. Effects of similar experimental approaches as being employed currently, that is, NOS inhibition in parallel with CYP treatment, have been reported previously in the literature [36, 37]. These studies, in which no functional parameter has been assessed, show that pretreatment with a NOS inhibitor prevents increased CYP-induced extravasation, bladder weight, and plasma nitrite-nitrate levels. In the mentioned studies, the NOS inhibition was administered only a few hours before the CYP treatment. In our pilot experiments, trying to determine suitable doses, the pretreatment was administered close to the CYP injection. However, no effect could be then seen on the CYP-induced functional alterations. Consequently, a longer pretreatment dosage regime was employed. Anyhow, the differences in the results of the pretreatment indicate that inflammatory changes do not all have the same sensitivity towards pharmacological modulation of the NO system.

The results of the current study are in accordance with what is mentioned in the introduction. Namely that, in the rat, concomitantly to a mucosal NOS upregulation, muscarinic M5 receptors are upregulated 60 hours after the CYP treatment [6], which has been suggested to be involved in the urothelial/mucosal production of NO. Noteworthy, even though this is not the only source for NO production [38], it seems to dominate the muscarinic coupled release [14]. Furthermore, P1A1 purinoceptors, suggested to exert proinflammatory effects, show pronounced plasticity and, based on experiments on urothelial cell lines, seem to be downregulated during the course of inflammation [39]. This phenomenon is supported by observations made in the rat, in which P1A1 purinoceptors in the urinary bladder are downregulated 60 h after CYP treatment [32]. As also mentioned above, muscarinic receptor-induced contractions to methacholine are reduced after CYP treatment, and, further, the contractile impairment is greater than what could be explained by a muscular insufficiency only [40]. However, the major part of the contractile decrement depends on a general alteration of the tissue responsiveness.

Before the effectuation of the current study, the observations mentioned above led us to a tentative standpoint for how the bladder inflammatory key molecules interact. Namely, an increased formation and release of purines and of adenosine in particular enhance an inflammatory defense including stimulation of the release of acetylcholine of nonneuronal origin [15]. The acetylcholine induces an upregulation of NOS and the release of NO that aggravates the condition. During the course of inflammation, the P1A1 purinoceptors are downregulated, which may seem logical in view of protecting the tissue from negative effects of an exaggerated inflammatory response. The inflammatory alterations cause functional disturbances, which may be counteracted by muscarinic antagonists or NOS inhibitors [6], as well as by P1A1 purinoceptor antagonist [31]. But is this the only effect of the substances? Can they even prevent the development of the condition into an aggravated state?

In the current study, the results of the pretreatment with the NOS inhibitor were clear-cut regarding functional responses, showing that pretreatment with L-NAME tended to normalize the responses in the CYP-treated animals. This may be particularly obvious when considering the responses close to the EC_50_. In the case of 4-DAMP, a dose of 1 mg/kg was used, which possibly may have been too low since the blockade of muscarinic receptors had minor or no direct effect on the inflammatory process. However, in similar experiments doses of other antagonists within this range have proven to be effective [31]. Also, the general reduction of methacholine-evoked responses may be an effect of a remaining 4-DAMP blockade, suggesting a sufficient dose. Both pretreatments (4-DAMP and L-NAME) seemed to reduce the contractility in the saline-treated animals, which was particularly obvious when analyzing the ATP contractile responses. However, ATP responses seem to be less robust than methacholine-evoked responses. One reason to this is of course the magnitude of the ATP contractile responses, which is substantially smaller than the methacholine-evoked contractions. Nevertheless, in spite of the general pretreatment decrementing effect, the contractions in the L-NAME pretreatment group that had been CYP-treated seemed to respond better than any other CYP-treated group did. However, comparisons between the pretreatment groups should be made with caution. This is because saline and CYP treatments, rather than antagonist pretreatments, were performed in parallel; thus, comparisons between saline and CYP treated animals are more relevant. Generally, the ATP-evoked contractions tended to be reduced in the saline-treated rats, but not in the CYP-treated ones (particularly not in the L-NAME pretreated). Nevertheless, the reduction in the saline-treated bladders thwarts the interpretation of the data. Also, the adenosine relaxations were smaller in the 4-DAMP and L-NAME pretreated preparations in the saline-treated group. However, the L-NAME pretreatment revealed the same overall pattern for adenosine-evoked relaxations as for methacholine-evoked contractions.

The assumption of the upstream urothelial muscarinic M5 receptor regulation of NO inflammatory effects now has to be rephrased. The reported effect on NO mechanisms by muscarinic receptor blockade, probably occur in the acute micturition situation. In the course of inflammation, the muscarinic receptor mechanism seems to be of less importance. Part of the stimulus for NO formation has to be searched for elsewhere. Importantly, recent studies confirm that increased levels of NOS are of great importance in the development of cystitis, regardless of experimental procedure, making this enzyme a fundamentally important pharmacological target [41]. It has also previously been demonstrated that interferon gamma, among other factors, can increase the expression of inducible NOS in the rat, which may constitute a nonmuscarinic NOS stimulation [42]. The morphological signs in the current study of the cystitis included an upregulation of muscarinic M5 receptors and possibly a downregulation of P1A1 purinoceptors, which confirm previous findings [6, 32, 40]. In agreement with the effects of the pretreatment on the functional responses, 4-DAMP pretreatment had no effect on receptor expression, while L-NAME pretreatment prevented the upregulation of muscarinic M5 receptors and possibly the downregulation of the P1A1 purinoceptors in cystitis.

The morphological correlations to the functional changes were also sought for in bladder wall changes, since one reason for the general alteration of the bladder tissue responsiveness in the CYP-treated rats may be found in the disorganization of the smooth muscle layer. Concerning the total thickness, a small increase might have occurred by the 4-DAMP and L-NAME pretreatments in the saline-treated animals, while no effect of the pretreatments could be observed in the CYP-treated animals. However, the examination of mucosal thickness revealed that L-NAME pretreatment prevented CYP-induced changes. In this context it is interesting to consider the small, but not significant, reduction that may have occurred in the CYP-treated animals that were pretreated with 4-DAMP. Namely, as mentioned above, it has previously been demonstrated that there exists a link between muscarinic receptor stimulation and NO effects in the CYP-treated rat [6], which may play some minor role even for structural inflammatory effects. The present results thus support an upstream muscarinic receptor regulation of NO production to be important for some inflammatory changes in the bladder in the rat.

In order to evaluate how severe the CYP-induced cystitis may be, the occurrence of mast cells in the detrusor muscle was counted. The number of mast cells was fairly the same outside the detrusor smooth muscle layer in the different pretreatment groups. In the healthy rat bladder almost no mast cells occurred in the muscle layer, whereas in the CYP-treated rats, a small, but definite number appeared in the muscle layer. This corresponds to what has been reported in BPS/IC in humans [43]. The effects of the pretreatment with 4-DAMP and L-NAME showed the same pattern on the mast cell distribution as it did on functional responses and on receptor expression. That is, while 4-DAMP had no effect, L-NAME eliminated the mast cell appearance in the detrusor muscle in cystitis.

In contrast to the L-NAME effect on function, muscarinic M5 receptor expression, P1A1 purinoceptor expression, mast cell detrusor occurrence, and mucosal enlargement, L-NAME did not affect the MIF expression. It showed an identical occurrence after 4-DAMP and L-NAME pretreatments as after saline pre-treatment both in the saline and in the CYP groups. This underlines that NO cannot be the only mediator of CYP-induced changes. Other key molecules in the pathogenesis are purines, in particular adenosine [31]. Obviously and rather expectedly the pathogenesis is orchestrated by a number of participating molecules of which NO is only one.

## 5. Conclusions

The current study shows that pretreatment with a NOS inhibitor reduces many of the changes that are induced by CYP-treatment. A muscarinic receptor antagonist, on the other hand, seems to have little effect on the development of the CYP-induced cystitis in the rat. While NO is involved both in the cystitis development and in the direct function impairment, the urothelial muscarinic receptors that via NO regulate part of the hampered bladder contractility during CYP-induced cystitis seem to be less important in the long-term (indirect) regulation of function and morphology during cystitis. Thus, while there exists an urothelial muscarinic receptor-NO coupling in the control of function in cystitis [40], this coupling seems to be of minor importance in the regulation of inflammation development in the rat urinary bladder. In general, the data indicate that urothelial factors, such as NO, affect not only the sensory signaling, but also mucosal morphology as well as detrusor contractility.

## Figures and Tables

**Table 1:** Methacholine concentration-response curve characteristics. Nonlinear analyses according to bell-shaped response-curves.

Treatment	*n*_H_ (first part of biphasic curve)	log⁡EC_50_	*R* ^2^	Curve differences (*F*, *P*)
Saline-saline	0.74	−4.80	0.71	4.38, 0.001
Saline-CYP	0.89	−5.43	0.75	
4-DAMP-saline	0.75	−4.13	0.85	7.26, <0.0001
4-DAMP-CYP	0.89	−5.25	0.64	
L-NAME-saline	0.91	−5.00	0.79	2.55, 0.035
L-NAME-CYP	0.97	−5.41	0.83	

**Table 2:** Receptor expression and mast cell occurrence as judged by muscarinic M5 receptor-like, P1A1 purinoceptor-like and MIF-like staining in the detrusor muscle in saline and CYP-treated rats that had received saline, 4-DAMP, or L-NAME pretreatment (*n* = 5 in each group). 0, (+), +, ++ refer to none (or almost none), possibly occurring, occurring, and obvious, respectively.

	Muscarinic M5 receptor-like staining	P1A1 purinoceptor-like staining	MIF-like staining
Saline-saline	+	+	(+)
Saline-CYP	++	(+)	++
4-DAMP-saline	+	+	(+)
4-DAMP-CYP	++	(+)	++
L-NAME-saline	+	+	(+)
L-NAME-CYP	(+)	+/(+)	+

## Key of Definitions for Abbreviations

4-DAMP: 4-diphenylacetoxy-N-methylpiperidine methiodide
ATP: Adenosine-5′-triphosphate
BPS: Bladder pain syndrome
CYP: Cyclophosphamide
IC: Interstitial cystitis
L-NAME: N_*ω*_-nitro-L-arginine methyl ester
NO: Nitric oxide
NOS: Nitric oxide synthase.
